# Comparison of 24-hour versus random urine samples for determination and quantification of Bence Jones protein in a South African population

**DOI:** 10.4102/ajlm.v10i1.1228

**Published:** 2021-08-04

**Authors:** Ashandree Reddy, Nadine Rapiti, Verena Gounden

**Affiliations:** 1 Department of Chemical Pathology, Faculty of Health Sciences, University of KwaZulu-Natal, Durban, South Africa; 2 National Health Laboratory Service, Durban, South Africa; 3 Department of Haematology, Faculty of Health Sciences, University of KwaZulu-Natal, Durban, South Africa

**Keywords:** multiple myeloma, Bence Jones protein, random urine, estimated 24-h Bence Jones protein

## Abstract

**Background:**

The International Myeloma Working Group and College of American Pathologists recommend a 24-h urine collection to determine the Bence Jones protein (BJP) excretion level for monitoring treatment response in patients with multiple myeloma (MM). There are several issues related to sample collection and the method is prone to inaccuracy.

**Objective:**

This study compared measured 24-h to random urine collections for the quantitation of BJP in a South African population.

**Methods:**

Sixty-six patients with MM submitted random urine samples with their routine 24-h urine collection from April 2016 – March 2018. Measured 24-h urine BJP was compared to two estimated 24-h BJP excretions calculated as follows: Estimation 1 (E1): Estimated 24-h BJP (mg/24 h) = Urine BJP/Creatinine ratio (mg/mmol) × 10. Estimation 2 (E2): Estimated 24-h BJP (mg/24 h) = Urine BJP/Creatinine ratio (mg/mmol) × 15 mg/kg for women or × 20 mg/kg for men.

**Results:**

Correlation of estimation equations E1 and E2 to the measured 24-h urine BJP was 0.893. Patients showed no difference in classification of treatment response using either the E1 or E2 estimation equations when compared to the measured 24-h urine BJP results.

**Conclusion:**

This study demonstrates that the estimated 24-h BJP shows a high degree of correlation with the measured 24-h BJP and can likely be used to monitor treatment response in South African patients with MM.

## Introduction

Plasma cell neoplasms are a group of disorders which include multiple myeloma (MM), where a clone of plasma cells can secrete a homogenous immunoglobulin or its components. These may be identified as a monoclonal peak on analysis by serum or urine protein electrophoresis (UPEP).^1^ The presence, level and type of monoclonal immunoglobulin have important implications in diagnosis, staging and treatment of these disease states.^2^

The core diagnostic features of MM include the presence of neoplastic plasma cells on bone marrow aspirate, radiological evidence of osteolytic lesions and detection of monoclonal immunoglobulin in serum or urine.^1^ It is the second most common haematological cancer accounting for 1% of all malignancies worldwide. Multiple myeloma was responsible for 0.43% of newly diagnosed cases of malignancies in South Africa in 1999 with the incidence being reported at approximately 0.00054%.^3^ While the incidence is highly variable among countries, studies indicate that the incidence of MM has increased uniformly since 1990 with the largest increase in middle and low-middle income countries.^4,5^ The prevalence of MM is higher in HIV-positive compared to uninfected individuals.^6^ This increases the disease burden of MM in South Africa, a country that has a high HIV epidemic prevalence with 7.5 million people living with HIV.^7^

Monoclonal free light chains (FLCs) appearing in the urine are referred to as Bence Jones proteins (BJPs). Detection and measurement of BJPs are utilised to diagnose and monitor monoclonal gammopathies.^8,9^ Once renal tubular reabsorption is saturated, BJP is present in urine. In approximately 20% of MM cases, BJPs may occur in the absence of a monoclonal band in the serum, making their detection a valuable test for this malignancy.^8,10^

Levels of BJPs may be quantified by UPEP. Following electrophoresis of the urine specimen and staining of the gel, the size of the BJP peak is measured using a densitometry scan of the peak. The percentage area of the peak is then multiplied by the total urine protein concentration of the sample to provide a semi-quantitative value for the BJP. Confirmation of the presence of BJP following UPEP is performed via immunofixation.

The International Myeloma Working Group (IMWG) and the College of American pathology recommend a 24-h urine collection for quantification of urine BJP.^9,11^

Although 24-h urine is a definitive means to determine renal protein excretion, it has several issues especially those related to sample collection (Table 1)^12,13,14,15^. In particular, the impracticality of a 24-h collection together with the high likelihood of incomplete collections hinder the accuracy of the test. Hence the use of random or early morning urine collections has been suggested to avoid the problems associated with 24-h collections. The clinical utility of measured urine protein is improved when expressed as a ratio to urine creatinine.^12,13,14^ As creatinine excretion in urine is fairly constant throughout the 24 h, measurement of protein creatinine ratios allows correction for variations in urine concentration. The use of protein creatinine ratios has become widespread for routine urine protein analysis and several studies have demonstrated good correlation with the 24-h collection.^11,12,13^

**TABLE 1:** Comparison of the advantages and disadvantages of random and 24-h urine collection for Bence Jones protein.

Method	Advantages	Disadvantages
Random urine	Easy to obtain Rapid transfer to the laboratory which avoids potential degradation Collected any time of day No need for the patient to store sample and transport to the laboratory	Synthesis and release of Bence Jones protein may be variable throughout the day
24-h urine	Defines Bence Jones protein excretion over the entire 24 h Directly relates to published data on 24-h Bence Jones protein excretion	Inconvenient and complex for patient collection, storage and transport to the hospital Frequent incomplete collections Total urinary protein varies with urine volume

*Source*: See full reference list of the article Beetham R, Cattell WR. Proteinuria: Pathophysiology, significance and recommendation for measurement in clinical practice. Ann Clin Biochem. 1993;30(5):425–434. https://doi.org/10.1177/000456329303000502, for more information.

While the use of BJP to creatinine ratio has emerged as an alternative to the 24-h collection, few studies have examined its correlation with the 24-h collection and no reported study to the authors’ knowledge has reviewed its utility in an African population.^14,15,16,17^

The haematology clinic at King Edward VIII Hospital is the referral centre for the management of patients with MM and other plasma cell neoplasms from the entire province of KwaZulu-Natal, South Africa. Many of these patients carry their 24-h collections, travelling several hundred kilometres using public transport to reach the haematology clinic. This is not ideal for maintaining sample stability while also being inconvenient and embarrassing for the patient.^18^

Despite new therapy options, MM is largely incurable, and most patients relapse and require a change in management. Laboratory testing plays a vital role in monitoring response to treatment as well as detecting a relapsed inpatient on treatment.^9^ This, together with the previously described issues related to 24-h urine collections, prompted us to examine the utility and validity of measured 24-h urine compared to random urine collections for the quantitation of BJP in a South African population.

## Methods

### Ethical considerations

Ethical approval to conduct the study was acquired from the Biomedical Research Ethics Committee, University of KwaZulu-Natal (ref. no. BE509/15). Written informed consent was taken from each participant in English or isiZulu depending on their requirement. The raw data were securely stored by the principal investigator and the compiled electronic data were anonymised and password protected for use only by those involved in the study.

### Participants

Study participants were recruited from the haematology clinic at King Edward VIII Hospital, Durban, South Africa. Samples were collected over a period of two years (April 2016 – March 2018). All participants had the diagnosis of MM (per IMWG criteria) and were at different stages of the disease and treatment.

### Sample collection

Each participant collected a 24-h urine sample for BJP following a standard protocol as part of the routine clinical assessment. The 24-h collection was started the day before the clinic visit. On submission of the 24-h collection, the participants immediately collected a random urine sample as per instructions provided. Thymol was used as the preservative for the 24-h urine sample and no preservative was utilised for the random sample. Both samples were submitted to the laboratory immediately. The 24-h collections were analysed as per routine by the chemical pathology laboratory.

The random urine samples were analysed for urine total protein (UTP) and creatinine. Aliquots of the random urine samples were then frozen at –70 °C and stored for a maximum of one month (stability as per manufacturer) until the UPEP was performed.^18^

### Laboratory analysis

For both random and 24-h urine collections, UPEP was performed using the Sebia Hydragel 7 high resolution kit run on the Sebia Hydrasys (Sebia, Norcross, Georgia, United States). Quantitation of UPEP fractions was performed using the Sebia Hydrasys densitometer system and Phoresis software (Sebia, Norcross, Georgia, United States). Acid violet staining was used, and the sensitivity of this method allows BJP to be detected at concentrations of 15 mg/L – 20 mg/L of the original urine. Urine samples for immunofixation electrophoresis (IFE) analysis were concentrated using BJP concentrators from the Sebia Hydrasys kit for all UTP samples measuring less than 0.7 g/L.^18^ Urine total protein and urine creatinine were measured using standard spectrophotometric methods on the Siemens ADVIA 1800 chemistry analyser (Siemens Diagnostics, Tarrytown, New York, United States). A dye-binding method using pyrogallol red was used to quantify UTP.^19^ Urine creatinine was measured using the modified kinetic Jaffe method.^20^

Only those 24-h urine samples that were positive for BJP had their respective random samples analysed to determine comparability. The measured 24–h BJP excretion was calculated as follows: %BJP peak on densitometer × UTP (g/L) × 24-h urine volume (litre [L]) and multiplied by 1000 mg/24 h. The estimated 24 BJP using the random urine values were calculated as per the two formulae below:

$$\begin{array}{l} Estimation\;1\;(E1):\text{Estimated 24-h BJP }(\text{mg / 24 h})=\\ \text{Urine BJP / Creatinine ratio }(\text{mg / mmol})\times 10 \end{array}$$ [Eqn 1]

For E1, a factor of 10 was utilised because while daily excretion of creatinine is dependent on muscle mass, an average daily loss of 10 mmol of creatinine can be expected.^13^

$$\begin{array}{l} Estimation\;2\;(E2):\text{Estimated 24-h BJP }(\text{mg / 24 h})=\\ \text{Urine BJP / Creatinine ratio }(\text{mg / mmol})\times 15\text{ mg/kg}\\ \text{for women or}\times 20\text{ mg/kg for men }(\text{convert mg/kg}\\ \text{to mmol/kg by multiplying }0.00884) \end{array}$$ [Eqn 2]

E2 was based on the average urinary creatinine excretion which is higher in men (14 mg/kg/day – 26 mg/kg/day) than women (11 mg/kg/day – 20 mg/kg/day).^13^

Of the included participants, three had paired before and after treatment samples. For the purpose of this study, they were classified according to IMWG response criteria^21^ using only the urine and serum electrophoresis data. A complete response is a negative serum and urine M-protein immunofixation; a very good partial response is serum and urine M-protein detectable by immunofixation but not by electrophoresis or a greater than 90% reduction in serum M-protein plus urine M-protein level under 100 mg/24 h; a partial response is a greater than 50% reduction in serum M-protein and a greater than 90% or to under 200 mg/24 h reduction in 24 h urine M-protein; progressive disease is described as an increase of greater than 25% from the lowest response value in any one or more of the following: (1) serum M-component or the absolute increase must be over 0.5 g/dL, (2) Urine M-component and/or the absolute increase must be > 200 mg/24 h. Stable disease is not meeting criteria for complete response, very good partial response, partial response or progressive disease.^21^

Demographic details and clinical histories were collected from the patients’ clinical records. The body mass index was calculated as weight/height^2^ (kg/m^2^) and categorised according to the World Health Organization.

### Statistical analysis

Statistical analyses were performed using Microsoft^®^ Excel (Microsoft^®^ Office 2016, Microsoft, Redmond, Washington, United States) and MedCalc for Windows, version 10.0 (MedCalc Software, Ostend, Belgium). The Shapiro-Wilk test was used to assess normality. For non-parametric data, the Spearman rank correlation and Passing-Bablock regression analysis was utilised for comparison of different estimated 24-h BJP equations to the measured 24-h BJP. Categorical data were compared using the Kruskal Wallis test. Wilcoxon paired-sample analysis was used to compare continuous variables. A *p*-value of less than 0.05 was deemed to be statistically significant.

## Results

A total of 66 paired 24-h and random urine samples were collected. Twenty-two samples (33%) had detectable BJP on 24-h UPEP and 19 (29%) had a quantifiable BJP in g/24 h. Three patients had faint bands below the detectable limit (< 15 mg/L) on the measured 24-h urine with percentage BJP calculated, but did have a quantifiable BJP peak on their paired random urine sample. The urine total protein (TP) for those three 24-h samples were 0.1 g/L, 0.07 g/L and 0.05 g/L which was much lower than their random paired samples of 1.6 g/L, 1.1 g/L and 1.8 g/L. One sample had a quantifiable 24-h BJP peak (TP 0.1 g/L) with no peak on the random urine sample (TP 4.2 g/L) but the monoclonal band was present on urine immunofixation.

The 22 samples with detectable BJP were from 19 patients as three patients had repeat collections within the study period. For the three patients, only their first collection was included when analysing data except when categorising the responses. There were 10 women and 9 men with 18 of the 19 patients being black African. The remaining patient was of Indian descent. Only 10 patients were tested for HIV with only 1 being positive. Serum free light chains (SFLCs) were also only measured in seven of these patients. Of the 19 patients, two had no urine immunofixation request by a pathologist or analysis despite having detectable BJP on UPEP (Table 2). The mean age was 55.8 years old (standard deviation ± 6.6) and the mean body mass index was 27.5 m^2^/kg (standard deviation ± 5.4) (Table 3).

**TABLE 2:** Patient characteristics including demographics, immunotyping and relevant laboratory results obtained from King Edward Hospital, Durban, South Africa, April 2016 – March 2018.

No.	Age	Gender	Serum immunofixation electrophoresis	Urine immunofixation electrophoresis	Bence Jones protein (mg/24 h)	E1 (mg/24 h)	E2 (mg/24 h)	Total serum calcium (mmol/L)	Albumin (g/L)	Estimated glomerular filtration rate (mL/min/1.73m^2^)	Total protein (g/L)	Haemoglobin (g/dL)	Serum free light chain ratio
1	58	F	IgA K and free K	Free K	2520	1256.28	949.52	2.65	42	17	81	5.2	ND
2	74	M	IgG K and free K	n/a	1080	1286.17	1705.47	2.03	22	15	98	5.9	ND
3	57	F	IgG K and free K	Free K	3430	1636.36	1562.27	1.89	17	> 60	41	5.7	ND
4	61	M	IgG K and free K	Free K	690	899.60	795.25	2.06	16	> 60	108	7.7	ND
5	59	F	IgG K and free K	Free K	< 0.15	350.82	437.27	2.20	26	24	82	9.3	ND
6	54	F	IgA K and free K	IgA K and free K	6800	4712.54	4499.15	1.70	20	7	86	7.7	ND
7	59	M	IgA K	Free K	< 0.15	460.51	447.80	2.17	29	36	95	8.6	4.73
8	58	M	IgG K	IgG K	380	597.97	1046.63	3.18	20	18	153	6.4	ND
9	63	M	IgG K	n/a	< 0.15	397.14	575.76	2.65	28	37	100	8.8	ND
10	47	M	Free L	Free L	14 400	7142.86	9597.71	3.36	34	16	73	5.9	ND
11‡	56	F	Free L	Free L	8580	5137.11	5790.04	2.60	40	48	73	13.3	0.01
12	53	F	IgG L and free L	IgG L and free L	380	393.55	412.26	2.20	35	19	83	9.1	ND
13	65	M	Free K	Free K	2660	1917.79	2034.39	2.28	40	> 60	67	7.9	1.76
14	57	F†	IgG K	IgG K and free K	250	0.00	0.00	1.90	19	9	96	7.4	ND
15‡	47	F	IgA K	IgA K and free K	2480	4481.63	3743.86	3.49	16	8	140	6.7	102.23
16‡	49	M	IgA K	IgA K and free K	680	211.84	269.67	2.04	21	50	109	9.2	0.76
17	49	F	IgA L	Free L	1200	1628.22	1403.36	2.60	21	23	118	6	ND
18	55	M	Free L	Free L	3600	956.73	1945.23	2.19	38	> 60	65	10.3	0.01
19	56	F	Free K	Free K	6150	7087.63	6484.76	2.59	37	10	72	6.6	leaked
Mean (s.d.)					2500	2362.00	2629.00	2.4 (0.5)	27.4 (8.9)	10.0	91.6 (25.9)	7.8 (1.9)	-

Reference ranges: serum free light chain 0.26–1.65, calcium 2.15 mmol/L – 2.55 mmol/L, albumin 35 g/L – 52 g/L.

F, female; K, kappa; L, lambda; M, male; ND, not done; s.d., standard deviation; Ig, immunoglobulin; n/a, not applicable.

† , Patient of Indian decent;

‡ , The three patients are those with samples before and after treatment. Only their initial samples are included in this table.

**TABLE 3:** Summary data of the patient characteristics, the mean or median for the measured 24-h Bence Jones protein and the Estimated Bence Jones protein for the 19 patients, between April 2016 and March 2018, Durban, South Africa.

Parameter	Mean ± s.d.	Median	Range
Age (years)	55.8 ± 6.6	-	-
Body mass index (m^2^/kg)	27.5 ± 5.4	-	-
Measured 24-h Bence Jones protein (mg/24 h)	-	2500	250–14 400
E1 (mg/24 h)	-	2362	211–7143
E2 (mg/24 h)	-	2629	267–9597

Note: E1 refers to Estimated 24-h BJP (mg/24 h) = Urine BJP/Creatinine ratio (mg/mmol) ´10 and E2 refers to Estimated 24-h BJP (mg/24 h) = Urine BJP/Creatinine ratio (mg/mmol)´ 15 mg/kg for women or × 20 mg/kg for men (convert mg/kg to mmol/kg by multiplying 0.00884).^13^

*N* = 19.

s.d., standard deviation.

The correlation between E2 and the 24-h BJP is 0.88 for women and 0.988 for men. The E2 equation was based on the average urinary creatinine excretion which is higher in men (14 mg/kg – 26 mg/kg per day) than women (11 mg/kg – 20 mg/kg per day). The correlation between E1 which is based on creatinine excretion and 24-h BJP is 0.82 for women and 0.975 for men (Table 4).

**TABLE 4:** Estimated equations (E1 and E2) and 24-h Bence Jones protein categorised according to gender between April 2016 and March 2018, Durban, South Africa.

Patient no.	E1 (mg/24-h)	E2 (mg/24-h)	24-h Bence Jones protein (mg/24-h)
**Women**			
1	1256.0	949.5	2520.00
2	1636.4	1562.3	3430.00
3	350.8	437.3	< 0.15
4	4712.5	4499.2	6800.00
5	5137.1	5790.0	8580.00
6	393.6	412.3	380.00
7	0.0	0.0	250.00
8	4481.6	3743.9	2480.00
9	1628.2	1403.4	1200.00
10	7087.6	6484.8	6150.00
Mean	2668.0	2528.2	3179.00
**Men**			
1	1286.2	1705.5	1080.00
2	899.6	795.3	690.00
3	460.5	447.8	< 0.15
4	598.0	1046.6	380.00
5	397.1	575.8	< 0.15
6	7142.9	9597.7	14400.00
7	1917.8	2034.4	2660.00
8	211.8	269.7	680.00
9	956.7	1945.2	3600.00
Mean	1541.2	2046.4	2610.00

Analysis following categorisation of the three patients with paired samples per IMWG BJP response criteria (Table 5) showed no significant difference in classification of treatment response using either the E1 or E2 estimation equations when compared to the measured 24-h urine BJP results. Absolute or percentage difference from Sample A (before treatment) and Sample B (after treatment) did not show PR which is characterised by a greater than 50% reduction of serum M-protein and reduction in 24-h urinary M-protein by over 90% or to under 200 mg/24 h. Nor did it show progressive disease with an increase of more than 25% from the lowest response value urine M-component (the absolute increase must be > 200 mg/24 h). The average period between Sample A and Sample B was nine months. The treatment comprised supportive and specific individualised therapy. The specific therapy included the use of alkylating agents such as cyclophosphamide or melphalan, immunomodulatory agents like thalidomide and steroids. All three patients were classified as stable disease as per the response criteria. The revised international staging system for MM is not affected by the difference between the measured and estimate equations for these three patients as BJP is not included in its criteria.

**TABLE 5:** Comparison of 24-h Bence Jones protein with the estimate equations (E1 and E2) in three paired patient samples before and after treatment over the April 2016 – March 2018 period in Durban, South Africa, together with their response classification.

Patient	Sample	Bence Jones protein (mg/24 h)	E1 (mg/24 h)	E2 (mg/24 h)	International Myeloma Working Group response criteria^21^
1	A	8580	5137.1	5790.0	-
	B	9240	5286.4	5888.2	-
	Difference (%)	7.1	2.8	1.6	Stable disease
2	A	680	211.8	269.7	-
	B	290	297.0	383.4	-
	Difference (%)	57.4	40.0	42.0	Stable disease
3	A	2480	4481.6	3743.9	-
	B	1610	549.5	728.6	-
	Difference (%)	35	88.0	81.0	Stable disease

Note: International Myeloma Working Group response criteria were reviewed by comparing percentage difference before treatment, Sample A, and after treatment, Sample B.

**FIGURE 1:**
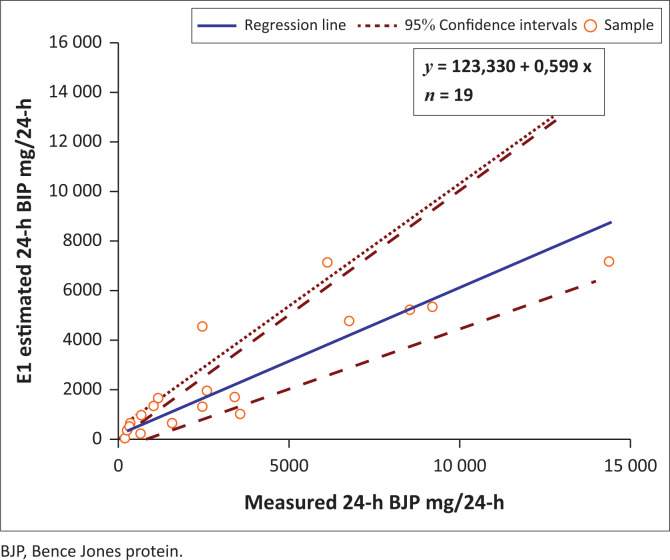
Regression analysis of measured 24hr Bence Jones protein excretion versus E1 estimation for 24-h Bence Jones protein during the period April 2016 – March 2018 in Durban, South Africa.

**FIGURE 2:**
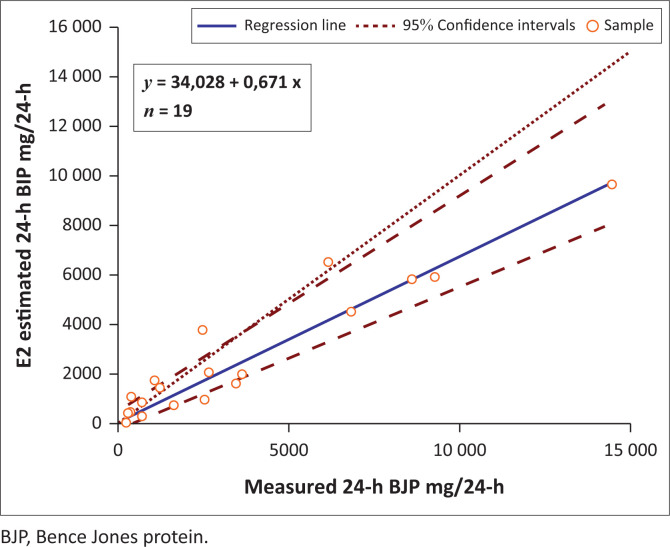
Regression analysis of measured 24-h Bence Jones protein excretion versus E2 estimation for 24-h Bence Jones protein, during the period April 2016 – March 2018 in Durban, South Africa.

The Spearman rank correlation for both estimation equations E1 and E2 was 0.893 when compared to the measured 24-h BJP. Passing-Bablock regression analysis showed that the E2 estimation equation had a smaller proportional bias with a slope of 0.968 compared to the E1 estimation equation slope of 0.671 when compared to the measured 24-h BJP (Figure 1 and Figure 2).

## Discussion

The Spearman rank correlation of 0.893 signifies a high degree of correlation between the estimate equations and 24-h BJP. This indicates that although the estimate equations do not precisely approximate the 24-h BJP, they are comparable and hence can be useful. The estimate equations also did not consistently overestimate or underestimate the 24-h measurement. The E2 estimation demonstrates a closer correlation and smaller proportional bias with the measured 24-h BJP compared to the E1 estimation equation and may be preferred (Table 4, Figure 1 and Figure 2).

Importantly, when the three paired samples’ estimated BJPs were used to classify patients according to IMWG treatment response, there was no significant difference with the performance of the measured 24-h BJP and the estimated BJP using the E1 and E2 equations (Table 5). This is key with regard to being able to use the random specimens for monitoring of disease. Although there was a limited number of samples, this study indicates the potential use of both 24-h BJP estimates to monitor response in patients with MM and this is in keeping with prior findings in other studies performed in different population groups.^16,17^

The average age of MM patients in this study was 55.8 years old, which is much younger when compared to that reported in developed countries with the average age at diagnosis ranging from 65 to 70 years.^22^ Due to the high prevalence of HIV infection in the 15–49-year-old age group (19%) in South Africa, further research with the appropriate clinical and laboratory information will be key in determining if HIV is a significant risk factor for MM in the younger population.^7^ Unfortunately this information was not available to us at the time. Other findings like translocation (11;14) have been reported to be more prevalent in younger myeloma patients.^23^

A previous study demonstrated that it may be possible to use the protein/creatinine ratio from random urine samples to estimate the 24-h BJP excretion.^16^ Another study concluded that protein concentrations in the same individual are relatively constant. This group also demonstrated that early morning spot specimens had a linear relation with measured 24-h BJP collections and were preferred over random urine collection.^17^ Because patients travelled long distances and arrived at the haematology clinic at varying times, early morning specimens were a challenge to collect. Despite this, our study was still able to demonstrate that a random sample can be comparable to a 24-h BJP and can be used to monitor disease response to treatment.

Light chains are more challenging to detect than complete Igs.^24^ The SFLC assay has increasingly been used and tracks well with proteinuria in individual patients.^25,26^ The greater sensitivity when compared to urine analysis has brought forth the widespread use and incorporation of SFLC measurements into multiple guidelines for the management of myeloma; most recently it is a myeloma defining event in asymptomatic patients.^9,27^ All the study participants had a serum protein electrophoresis and a UPEP but surprisingly only a few had SFLCs. We found only seven patients had SFLCs and one of the seven samples had leaked during transit. The SFLC assay is not readily available in our province (KwaZulu-Natal). Additionally, due to inter-patient variation in the renal metabolism of light chains, quantification of proteinuria cannot be predicted by the SFLC concentration.^28,29,30^ Fifteen of the 19 patients had glomerular infiltration rates under 60 mL/min per 1.73 m^2^ which may affect the renal metabolism of SFL’s. The IMWG states that once a diagnosis of MM is made, a 24-h UPEP and immunofixation should be done for patient monitoring and these measures are not replaceable with SFLC.^21,30^

Multiple myeloma is associated with significant mortality and morbidity and is considered largely incurable and fatal without treatment. With the introduction of new classes of effective drugs for the treatment of MM, improved frequencies and the degree of patient response have been observed. Many treatments have been shown to significantly prolong survival and simultaneously improve the quality of life. Unfortunately, all patients will ultimately relapse after treatment and will require a change to a more responsive therapy. This necessitates regular periodic monitoring of disease to detect relapse. Laboratory testing plays a vital role in monitoring response to treatment as well as detecting relapse in a patient on treatment.^9,21^ To further elucidate the findings in this study, future studies should include a larger cohort of patients that have measurements before and after treatment enabling a more robust comparison for response criteria.

This study is the first to use and to demonstrate the potential utility of the estimated 24-h urine BJP in an African population group. Both the E1 and E2 calculations are simple to perform while UTP and urine creatinine measurements are easily available on routine chemistry analysers.

Together with other studies, this study adds to the body of evidence available for use of random urine in estimating 24-h BJP for patient monitoring.^16,17^

### Limitations

As a result of only including patients with densitometrically quantifiable BJP on the measured 24-h BJP, the small sample size was a limitation. However, this was also a limitation noted in other studies reviewing the use of random urine collections for BJP estimation.^16,17^ Measuring the creatinine on the 24-h urine collections to verify the accuracy of the collection would have been beneficial.^17^ Another limitation is the challenges associated with the method to quantify BJP. Different proteins have varying affinities for the dyes used to stain electrophoretic gels and thus a lack of linearity of the densitometry response may be seen. Bence Jones protein may also co-migrate with other proteins or present with several bands making it complex to define the BJP peak correctly by densitometry. The measurement of BJP is not standardised and to minimise the mentioned analytical variability, it is suggested that patients should be followed up at the same laboratory, which was adhered to in this study.^31^ We suggest using the random BJP to monitor known patients with MM who already have confirmed BJP on immunofixation to minimise the above-mentioned limitations associated with measuring BJP on electrophoresis.

### Conclusion

The random urine BJP estimates E1 and E2 are simple, rapid, easily available and an inexpensive method that is comparable to a 24-h BJP. It can potentially be used for monitoring known patients with MM including light-chain disease. We have demonstrated that when using the IMWG response classification, both E1 and E2 equations did not differ from the measured 24-h BJP. Although we had a few paired samples, their encouraging comparison and utility will promote further studies with larger cohorts.
